# Iron(II) Complexes with Porphyrin and Tetrabenzoporphyrin: CASSCF/MCQDPT2 Study of the Electronic Structures and UV–Vis Spectra by sTD-DFT

**DOI:** 10.3390/ijms24087070

**Published:** 2023-04-11

**Authors:** Alexey V. Eroshin, Andrey I. Koptyaev, Arseniy A. Otlyotov, Yury Minenkov, Yuriy A. Zhabanov

**Affiliations:** 1 Research Institute of Chemistry of Macroheterocyclic Compounds, Ivanovo State University of Chemistry and Technology, Sheremetievskiy Av. 7, 153000 Ivanovo, Russia; alexey.yeroshin@gmail.com (A.V.E.); akisuct@gmail.com (A.I.K.); 2 Institute for Physics of Microstructures RAS, GSP-105, 603950 Nizhny Novgorod, Russia; 3 N.N. Semenov Federal Research Center for Chemical Physics RAS, Kosygina Street 4, 119991 Moscow, Russia; arseniy.otlyotov@chph.ras.ru (A.A.O.); yury.minenkov@chph.ras.ru (Y.M.); 4 Joint Institute for High Temperatures, Russian Academy of Sciences, 13-2 Izhorskaya Street, 125412 Moscow, Russia

**Keywords:** CASSCF, electronic structure, electronic spectra, iron(II) porphyrin, macroheterocycles

## Abstract

The geometry and electronic structures of iron(II) complexes with porphyrin (**FeP**) and tetrabenzoporphyrin (**FeTBP**) in ground and low-lying excited electronic states are determined by DFT (PBE0/def2-TZVP) calculations and the complete active space self-consistent field (CASSCF) method, followed by the multiconfigurational quasi-degenerate second-order perturbation theory (MCQDPT2) approach to determine the dynamic electron correlation. The minima on the potential energy surfaces (PESs) of the ground (^3^A_2g_) and low-lying, high-spin (^5^A_1g_) electronic states correspond to the planar structures of **FeP** and **FeTBP** with *D*_4h_ symmetry. According to the results of the MCQDPT2 calculations, the wave functions of the ^3^A_2g_ and ^5^A_1g_ electronic states are single determinant. The electronic absorption (UV–Vis) spectra of **FeP** and **FeTBP** are simulated within the framework of the simplified time-dependent density functional theory (sTDDFT) approach with the use of the long-range corrected CAM-B3LYP function. The most intensive bands of the UV–Vis spectra of **FeP** and **FeTBP** occur in the Soret near-UV region of 370–390 nm.

## 1. Introduction

Porphyrins and their metal complexes are promising materials for many high-technology industries, including the production of chemical sensors, photovoltaic devices, and microelectronic products.

Despite the fact that they were first obtained over 50 years ago [1], sufficiently stable synthetic iron(II) complexes with porphyrinoid ligands became available only decades later [2,3]. Since then, they have been intensively studied as components of many systems due to their remarkable properties.

Being analogs of the natural heme protein [2,4], these compounds exhibit high (photo)catalytic activity, especially in reactions involving oxygen [4,5,6]. On the one hand, the photosensitivity of the extended π-electron system of the macrocycle allows the use of iron porphyrins for light-harvesting devices [7]. On the other hand, due to the paramagnetic central atom, Fe(II) complexes have a non-zero spin and are promising candidates for building blocks in spintronic devices, ranging from molecular magnets to molecule-based spintronic and quantum information devices [8,9].

The value of the total spin strongly depends on the metal–ligand interaction. When switching from iron(II) porphyrin (**FeP**) to tetrabenzoporphyrin (**FeTBP**), the expansion of the π-electron system due to benzoannulation leads to an increase in the effective magnetic spin moment [8,9]. Note that the electronic structure of TBP is closer to that of porphyrin, not phthalocyanine [10]. However, to the best of our knowledge, no detailed examination of the electronic structures of these compounds has been performed to date.

In this work, an investigation of the geometry and electronic structure of iron(II) complexes with porphyrin and tetrabenzoporphyrin has been performed within the framework of the complete active space self-consistent field/multiconfigurational quasi-degenerate second-order perturbation theory CASSCF/MCQDPT2 methodology. The analysis of the electron density distribution was carried out in terms of the quantum theory of atoms in molecules (QTAIM). The electronic absorption spectra were simulated with use of the simplified TD-DFT method and compared to the experiment for **FeTBP**.

## 2. Computational Details

The electronic structures of **FeP** and **FeTBP** in ground and low-lying excited states have been studied with the use of the complete active space self-consistent field (CASSCF) method followed by multiconfigurational quasi-degenerate second-order perturbation theory (MCQDPT2) calculations accounting for the dynamic electron correlation. Six electrons in five molecular orbitals consisting mainly of the 3d orbitals of Fe atom were selected for the active space. The doubly occupied orbitals, corresponding to the 1s orbitals of C, N, and Fe as well as the 2s and 2p orbitals of Fe were frozen in the MCQDPT2 calculations. The singlet electronic states of both the **FeP** and **FeTBP** complexes were found to possess a complex composition (see Table S1) and the relative energies were more than 107.5 kJ/mol and 119.0 kJ/mol, respectively (Table 1). In contrast, the wave functions of the low-lying triplet (^3^A_2g_) and quintet (^5^A_1g_) states are single-determinant; therefore, density functional theory (DFT) methods can be directly applied for further calculations.

The geometry parameters of the *D*_4h_-structures of **FeP** and **FeTBP** corresponding to the minima on the PESs of the ^3^A_2g_ and ^5^A_1g_ electronic states were optimized using PBE0 functional and triple-zeta def2-TZVP basis sets [11] for all atoms taken from the EMSL BSE library [12,13,14].

All the above-mentioned calculations were performed with use of the Firefly QC package [15], which is partially based on the GAMESS (US) [16] source code.

The molecular models and orbitals demonstrated in electronic structure subsection were visualized by means of the Chemcraft program [17].

Electronic absorption (UV–Vis) spectra of **FeP** and **FeTBP** were simulated within the framework of the simplified time-dependent density functional theory (sTDDFT) approach [18] as implemented in the ORCA 5.0.3 software [19,20]. Long-range corrected CAM-B3LYP functional [21] and all-electron def2-TZVP basis sets [11] were employed for the single-point energy calculations on the PBE0/def2-TZVP optimized geometries. Auxiliary Coulomb fitting def2/J basis sets [22] were used in conjunction with the RIJCOSX approximation [23] to accelerate hybrid DFT computations utilizing tighter-than-default DEFGRID3 integration grid. All calculations were performed assuming triplet ground states of **FeP** and **FeTBP** and using unrestricted Kohn-Sham (UKS) determinants. The excitations up to 8 eV were considered. The solvent effects were taken into account using the solvation model based on density (SMD) approach [24] with the default parameters implemented for the DMF solvent. The results of the sTDDFT calculations were visualized with use of the Multiwfn 3.8(dev) program [25].

## 3. Results and Discussion

### 3.1. Electronic Structure

**Figure 1:**
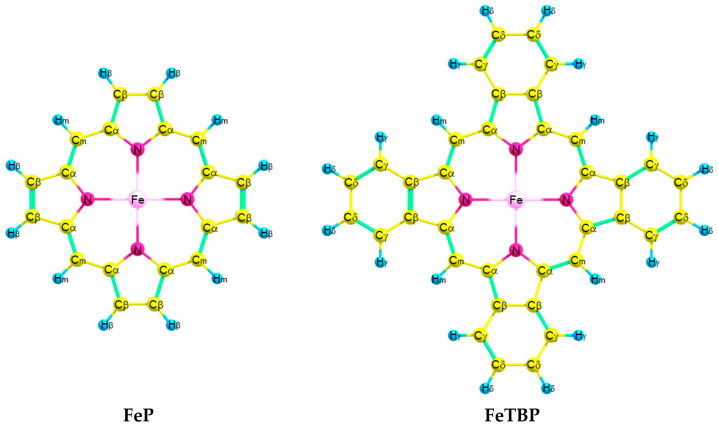
Molecular models of **FeP** and **FeTBP** with atom labeling.

The compositions of the wave functions for the ground and low-lying excited states of **FeP** and **FeTBP** are listed in Table S1. Table 1 contains the relative energies of excited states obtained from the MCQDPT2 calculations. Although according to the DFT calculations, the ^5^A_1g_ electronic state is lower in energy than ^3^A_2g_ (see Table 2) in the case of **FeTBP**, the MCQDPT2 method revealed that the ground state of both of the complexes **FeP** and **FeTBP** (Figure 1) is ^3^A_2g_. Note that DFT only considers dynamical correlation, whereas CASPT2 takes both dynamical and static correlations into account.

Analyzing Table S1, the complex composition of the low-lying singlet states wave functions of both metal complexes should be noted. In contrast, the wave functions of the ground states and the most low-lying high-spin states are single determinant. Hence, the geometry optimization along with force field and vibrational spectra computations were performed with use of the DFT method (at PBE0/def2-TVZP level) for the ^3^A_2g_ and ^5^A_1g_ electronic states. Table S1 shows that the unpaired electrons in the triplet (^3^A_2g_) are localized in doubly degenerated e_g_ orbitals, while for the low-lying ^5^A_1g_ state, the a_1g_ orbital is doubly occupied and the other active space orbitals are single occupied.

**Figure 2:**
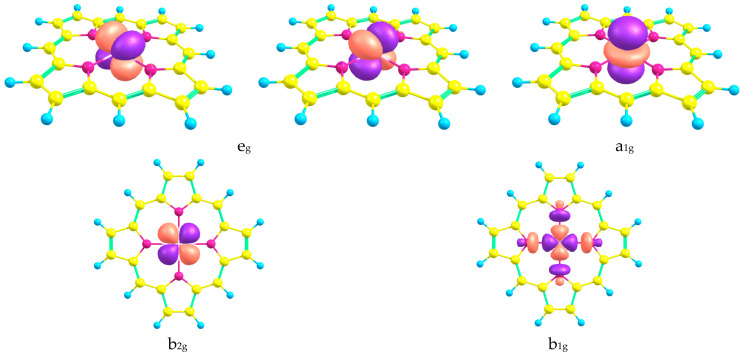
Shapes of active CASSCF molecular orbitals of **FeP**.

**Figure 3:**
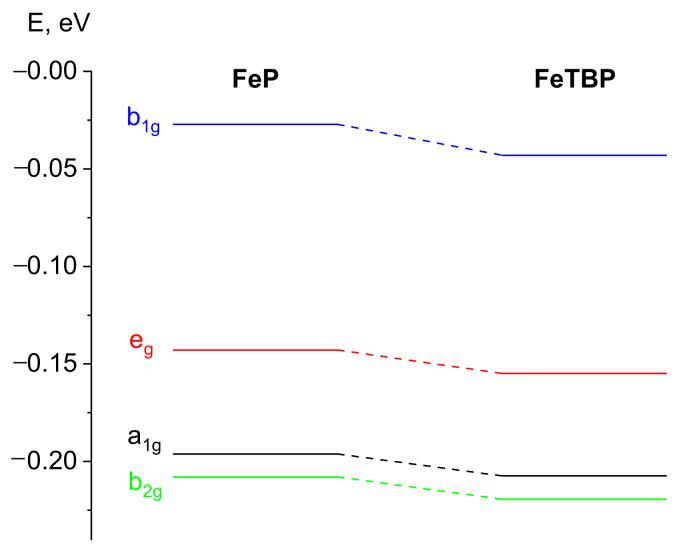
Energy diagram of the active in the CASSCF calculation’s molecular orbitals.

Orbitals, which mainly contain the 3d orbitals of the Fe atom, were included in the CASSCF active space (Figure 2). Herein, the contribution of the macrocyclic ligand atomic orbitals (AOs) is small except for the b_1g_ molecular orbital (MO) (see Figure 2). Consequently, the crystal field theory can be used for the sequence of electronic states describing. The energy diagram of the CASSCF active space MOs is demonstrated in Figure 3 and indicates the unfavorableness of the b_1g_ MO occupation. Meanwhile, the a_1g_, b_2g_, and e_g_ MOs possess remarkably lower energy; therefore, their occupation is preferable for **FeP** and **FeTBP**, similar to the iron(II) complex with porphyrazine **FePz** [26]. The occupation of the active in the CASSCF calculations orbitals and sequences of the electronic states of **FeP** and **FeTBP** indeed resembles that for complexes with a porphyrazine core ligand.

It should also be noted that the ^5^A_1g_ state of **FeP** is 24 kJ/mol higher in energy compared to the ground ^3^A_2g_ state (and only 8.5 kJ/mol higher in the case of **FeTBP**), while in the **FePz** [26], this difference is significantly larger and reaches ca. 105 kJ/mol. Therefore, the change of the nitrogen atom in the meso-position [26] by the CH group leads to the noticeable stabilization of the quintet state compared to the triplet; however, the latter still dominates.

### 3.2. Geometry Structure and Analysis of Electronic Density Distribution

According to our QC calculations, the investigated complexes **FeP** and **FeTBP** possess similar structures. Both molecules are planar and possess D_4h_ symmetry in their ground (^3^A_2g_) and lower high-spin (^5^A_1g_) electronic states.

The selected optimized structural parameters of these molecules are listed in Table 2.

The net atomic charges obtained by the PBE0/def2-TZVP approach for the ground and lower high-spin electronic states are presented in Table 3. It is worth mentioning that the use of the dispersion correction (PBE0-D3) [27] has almost no effect on the geometry: the largest difference does not exceed 0.002 Å and 0.1° for the internuclear bond distances and valence angles, respectively.

The size of the coordination cavity of the studied macrocycles can be described by the distances Fe–N, N…N, and C_m_…C_m_. These parameters are larger for the complex with tetrabenzoporphyrin **FeTBP** containing the fused phenyl rings. In contrast, the perimeter of the coordination cavity, defined as the sum of all N–C_α_ and C_α_–C_m_ bond distances, is slightly larger for **FeP** than for **FeTBP**. The reason for this discrepancy is the difference in the C_α_NC_α_ angle of the pyrrole rings. The significant elongation (~0.04 Å) of the C_β_–C_β_ bond length should also be noted in the case of **FeTBP** compared to **FeP**. Nevertheless, the net atomic charges for both complexes are almost the same. The largest difference was revealed for the C_β_ atoms with a minor negative charge. It has a higher absolute value in **FeP** due to the fact that the C_β_ atoms in this complex are bonded with hydrogens, while in **FeTBP,** they are slightly “robbed” by C_γ_ atoms.

Comparing the geometries of the ground (^3^A_2g_) and lower high-spin (^5^A_1g_) electronic states of both considered complexes, we should note the slight difference in the C–C and C–N bond distances except for C_α_–C_m_ parameter. For the latter, a noticeable (~0.01 Å) increase is observed in the quintet state, ^5^A_1g_, as compared to the triplet ground state, ^3^A_2g_. At the same time, the Fe–N distances are significantly longer in the case of the high-spin state. In addition, the net charge of the metal has a more positive value, while for nitrogen, it becomes more negative (see Table 3). Furthermore, the angle C_α_NC_α_ is also larger in the case of the ^5^A_1g_ states. The elongation of the Fe–N distance in the ^5^A_1g_ state is caused by the occupation of the antibonding b_1g_ MO (Figure 2).

Compared to iron porphyrazine **FePz** [26], an appreciable increase in the coordination cavity size is observed in **FeP**. For example, the Fe–N distance in **FeP** is longer by 0.08 Å in the ground state ^3^A_2g_. This can be related to a significant increase in C_α_X_m_ (X=C for **FeP** and X=N for **FePz**) as well as the C_α_X_m_C_α_ angle, since the C–C bond (in **FeP**) is longer than C–N (in **FePz**): 1.38 vs. 1.32 Å. Moreover, the bond distances of the pyrrole ring N–C_α_ and C_β_–C_β_ have almost the same values as in **FePz** [26], but the perceptible shortening of the C_α_– C_β_ bond is registered in **FeP**.

### 3.3. Electronic Absorption Spectra

**Figure 4:**
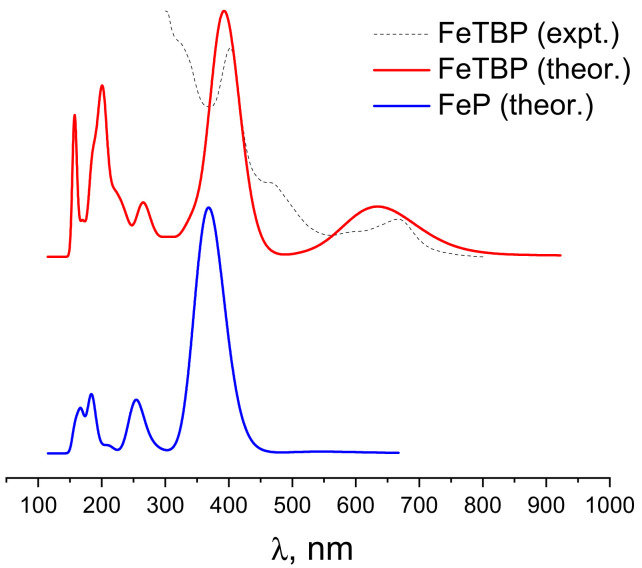
Normalized theoretical (sTDDFT) and experimental (for **FeTBP**) electronic absorption spectra. Gaussian broadening function with FWHM = 0.4 eV was used for the model spectra.

**Figure 5:**
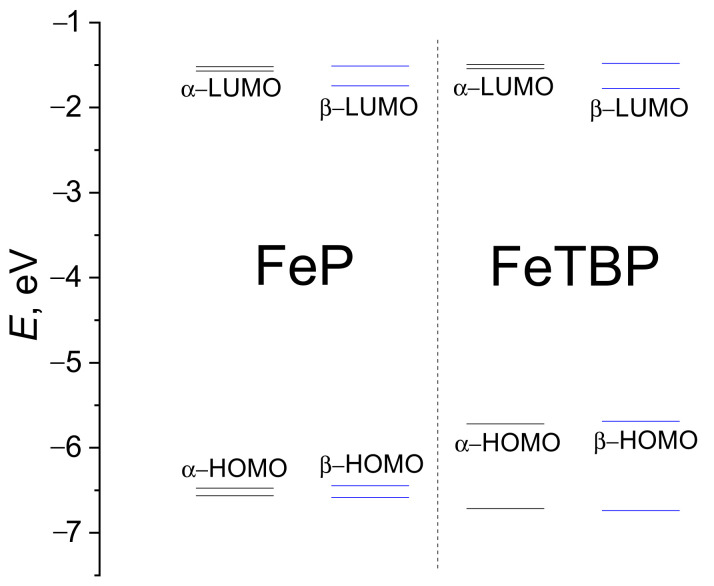
MO level diagram for **FeP** and **FeTBP** complexes according to UKS CAM-B3LYP/def2-TZVP calculations.

**Figure 6:**
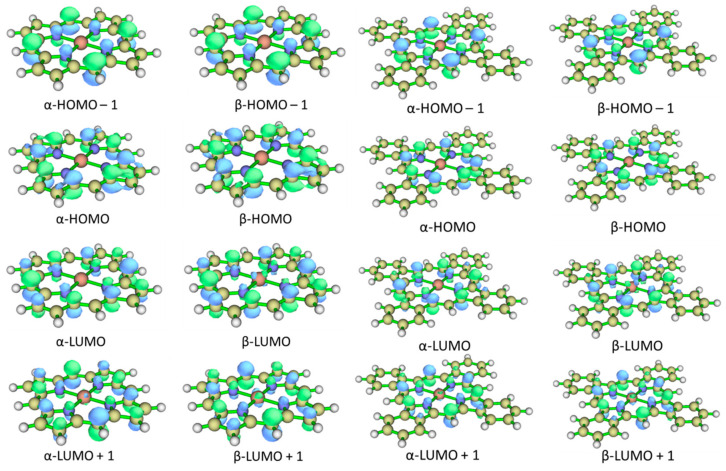
Shapes of the frontier molecular orbitals of **FeP** and **FeTBP** complexes.

According to the theoretical sTDDFT CAM-B3LYP SMD predictions (see Table 4), the most intensive bands of the UV–Vis spectra of **FeP** and **FeTBP** occur in the Soret near-UV region of 370–390 nm (see Figure 4). The corresponding excited states are formed by the electron transitions from the HOMO and HOMO–1 to LUMO and LUMO+1 orbitals (Figure 5 and Figure 6). The spectrum of **FeTBP** additionally contains the weaker Q-band at ca. 635 nm that is missing in the spectrum of **FeP**. The theoretical predictions for **FeTBP** are qualitatively supported by the experimental spectrum recorded in DMF. This also holds if other hybrid DFT functionals (PBE0 and B3LYP) are used for the simulation of the spectrum (see Figure S1). Moreover, the shapes of the occupied frontier MOs (HOMO and HOMO–1) are similar in **FeTBP** and the previously studied **ZnTBP** and **CdTBP** complexes [28].

## 4. Conclusions

An investigation of the geometry and electronic structure of **FeP** and **FeTBP** was performed. The minima on the potential energy surfaces (PESs) of the ground (^3^A_2g_) and low-lying high-spin (^5^A_1g_) electronic states correspond to the planar structures of **FeP** and **FeTBP** with *D*_4h_ symmetry. According to the data obtained by the MCQDPT2 method, the wave functions of ground (^3^A_2g_) and low-lying, high-spin (^5^A_1g_) electronic states have a single-determinant form, while a complex composition can be noted for singlet-state wave functions. The size of the coordination cavity depends on the electronic state and is larger in the quintet state for both complexes as compared to the ground triplet state, mainly due to the occupation of the antibonding b_1g_ orbital in the former. The applicability of the crystal field theory (CFT) for the description of electronic states sequences was demonstrated. The most intensive bands in the electronic absorption spectra of **FeP** and **FeTBP** occur in the Soret near-UV region of 370–390 nm and correspond to the electron transitions from the HOMO and HOMO–1 to LUMO and LUMO+1 orbitals. The simulated spectrum of **FeTBP** is in qualitative agreement with that of the experiment.

## Figures and Tables

**Table 1:** The relative energies (kJ/mol) of excited states from MCQDPT2 calculations.

State	ΔE, kJ/mol	State	ΔE, kJ/mol	State	ΔE kJ/mol	State	ΔE, kJ/mol
FeP		FeTBP		FeP		FeTBP	
^1^B_1g_	107.5	^1^B_1g_	119.0	^3^A_2g_	0	^3^A_2g_	0
^1^B_2g_	107.5	^1^B_2g_	120.6	^3^E_g_	15.8	^3^E_g_	11.1
^1^E_g_	116.2	^1^A_1g_	120.6	^3^B_2g_	79.8	^3^B_2g_	71.5
^1^A_1g_	117.1	^1^E_g_	123.1	^3^E_g_	137.7	^3^E_g_	132.6
^1^A_1g_	186.1	^1^A_1g_	194.6	^3^B_1g_	264.1	^3^B_1g_	249.1
^1^B_2g_	220.2	^1^B_2g_	224.1	^3^B_1g_	275.7	^3^E_g_	258.6
^1^E_g_	232.6	^1^E_g_	239.2	^3^E_g_	275.8	^3^B_1g_	259.7
^1^A_1g_	363.3	^1^B_1g_	348.1	^3^B_2g_	282.1	^3^B_2g_	266.1
^1^B_1g_	367.5	^1^A_1g_	351.1	^3^A_2g_	284.0	^3^A_2g_	267.0
^1^B_2g_	381.2	^1^E_g_	357.7				
^1^E_g_	382.5			^5^A_1g_	23.9	^5^A_1g_	8.5
				^5^E_g_	46.0	^5^E_g_	26.7
				^5^B_2g_	59.8	^5^B_2g_	42.8
				^5^B_1g_	290.6	^5^B_1g_	261.8

**Table 2:** Internuclear distances (Å), valence angles (degrees), and relative energies (kJ/mol) of the equilibrium D_4h_ structures calculated at PBE0/def2-TZVP level.

	FeP		FeTBP	
	^3^A_2g_	^5^A_1g_	^3^A_2g_	^5^A_1g_
Distances				
r(M–N)	1.990	2.054	2.014	2.077
r(N–C_α_)	1.366	1.361	1.367	1.362
r(C_α_–C_β_)	1.432	1.438	1.442	1.447
r(C_α_–C_m_)	1.380	1.392	1.375	1.386
r(C_β_–C_β_)	1.354	1.357	1.397	1.402
r(C_β_–C_γ_)	-	-	1.393	1.392
r(C_γ_–C_δ_)	-	-	1.380	1.381
r(C_δ_–C_δ_)	-	-	1.402	1.401
r(N…N)_opp_	3.980	4.108	4.028	4.154
r(N…N)_adj_	2.814	2.905	2.848	2.937
r(C_m_–C_m_)_opp_	6.800	6.835	6.810	6.843
r(C_m_–C_m_)_adj_	4.809	4.833	4.815	4.839
P ^a^	21.967	22.020	21.932	21.982
Angles				
∠(NC_α_C_m_)	125.4	125.2	125.7	125.6
∠(C_α_C_m_C_α_)	125.4	127.1	125.6	127.8
∠(C_α_NC_α_)	105.5	107.5	107.0	108.9
ΔE	0	38.4	7.1	0

^a^ Perimeter of the coordination cavity, see the text below.

**Table 3:** Net atomic charges (e) by PBE0/def2-TZVP calculations.

	FeP		FeTBP	
	^3^A_2g_	^5^A_1g_	^3^A_2g_	^5^A_1g_
N	−1.202	−1.254	−1.198	−1.249
C_α_	+0.438	+0.448	+0.438	+0.448
C_β_	−0.061	−0.063	−0.027	−0.028
H_β_	+0.063	+0.062	–	–
C_m_	−0.025	−0.033	−0.015	−0.024
H_m_	+0.052	+0.051	+0.045	+0.044
Fe	+1.186	+1.355	+1.192	+1.360
C_γ_	–	–	−0.027	−0.027
H_γ_	–	–	+0.042	+0.043
C_δ_	–	–	−0.034	−0.034
H_δ_	–	–	+0.043	+0.043

**Table 4:** Calculated compositions of the selected excited states of **FeP** and **FeTBP**, corresponding transition wavelengths, and oscillator strengths.

λ, nm	f	Composition (%)
**FeP**		
372.1	1.28	α-HOMO → α-LUMO + 1 (27) α-HOMO—1 → α-LUMO (25) β-HOMO → β-LUMO + 1 (25)
364.8	1.33	β-HOMO—1 → β-LUMO + 1 (29) α-HOMO—1 → α-LUMO + 1 (28) α-HOMO → α-LUMO (22)
**FeTBP**		
646.5	0.27	α-HOMO → α-LUMO (64) β-HOMO → β-LUMO (23) α-HOMO—1 → α-LUMO + 1 (7)
622.0	0.21	α-HOMO → α-LUMO + 1 (42) β-HOMO → β-LUMO + 1 (35) β-HOMO—1 → β-LUMO (17)
401.2	1.38	α-HOMO—1→ α-LUMO (51) β-HOMO—1 → β-LUMO (26) α-HOMO → α-LUMO + 1 (9)
383.3	1.18	β-HOMO—1 → β-LUMO + 1 (41) α-HOMO—1→ α-LUMO + 1 (38) α-HOMO → α-LUMO (7)

## Data Availability

The data presented in this study are available on request from the corresponding author.

## Supplementary Materials

The following supporting information can be downloaded at: https://www.mdpi.com/article/10.3390/ijms24087070/s1.
