# Supplementary material for: Iron(II) Complexes with Porphyrin and Tetrabenzoporphyrin: CASSCF/MCQDPT2 Study of the Electronic Structures and UV–Vis Spectra by sTD-DFT
Source: Int J Mol Sci. 2023 Apr 11;24(8):7070. doi: 10.3390/ijms24087070 (PMC10138890; doi:10.3390/ijms24087070)
Supplement: Supplementary file 1 [file ijms-24-07070-s001.zip › ijms-2303165-supplementary.pdf]

Supporting information for

Iron(II) complexes with porphyrin and tetrabenzoporphyrin: CASSCF/MCQDPT2 study of the electronic structures and UV-vis spectra by sTD-DFT

Alexey V. Eroshin, Andrey I. Koptyaev, Arseniy A. Otlyotov, Yury Minenkov and Yuriy A. Zhabanov

Content

Cartesian coordinates of $^1B_{1g}$ electronic state of FeP optimized PBE0/def2-TZVP level of theory:.............1

Cartesian coordinates of $^3A_{2g}$ electronic state of FeP optimized PBE0/def2-TZVP level of theory: 2

Cartesian coordinates of $^5A_{1g}$ electronic state of FeP optimized PBE0/def2-TZVP level of theory:.............4

Cartesian coordinates of $^1B_{1g}$ electronic state of FeTBP optimized PBE0/def2-TZVP level of theory: 5

Cartesian coordinates of $^3A_{2g}$ electronic state of FeTBP optimized PBE0/def2-TZVP level of theory: 8

Cartesian coordinates of $^5A_{1g}$ electronic state of FeTBP optimized PBE0/def2-TZVP level of theory: 10

Table S1. The relative energies (kJ/mol) of excited states and contributions (in %) of electronic configurations to the wave functions from MCQDPT2 calculations. 12

Figure S1. Normalized theoretical (sTDDFT) and experimental electronic absorption spectra of FeTBP in the DMF solvent. Gaussian broadening function with FWHM = 0.4 eV was used for the model spectra..14

Cartesian coordinates of $^1B_{1g}$ electronic state of FeP optimized PBE0/def2-TZVP level of theory:

**N**	**1.989715196900**	**0.000000000000**	**0.000000000000**
**N**	**0.000000000000**	**1.989715196900**	**0.000000000000**
**N**	**-1.989715196900**	**0.000000000000**	**0.000000000000**
**N**	**0.000000000000**	**-1.989715196900**	**0.000000000000**
**C**	**2.403266822600**	**2.403266822600**	**0.000000000000**
**C**	**-2.403266822600**	**2.403266822600**	**0.000000000000**
**C**	**-2.403266822600**	**-2.403266822600**	**0.000000000000**
**C**	**2.403266822600**	**-2.403266822600**	**0.000000000000**
**C**	**2.816971600100**	**1.086917445400**	**0.000000000000**
**C**	**-1.086917445400**	**2.816971600100**	**0.000000000000**
**C**	**-2.816971600100**	**-1.086917445400**	**0.000000000000**

C	1.086917445400	2.816971600100	0.000000000000
C	-2.816971600100	1.086917445400	0.000000000000
C	-1.086917445400	-2.816971600100	0.000000000000
C	2.816971600100	-1.086917445400	0.000000000000
C	1.086917445400	-2.816971600100	0.000000000000
C	4.191046876100	0.676242483400	0.000000000000
C	-0.676242483400	4.191046876100	0.000000000000
C	-4.191046876100	-0.676242483400	0.000000000000
C	0.676242483400	4.191046876100	0.000000000000
C	-4.191046876100	0.676242483400	0.000000000000
C	-0.676242483400	-4.191046876100	0.000000000000
C	4.191046876100	-0.676242483400	0.000000000000
C	0.676242483400	-4.191046876100	0.000000000000
H	3.169276985700	3.169276985700	0.000000000000
H	-3.169276985700	3.169276985700	0.000000000000
H	-3.169276985700	-3.169276985700	0.000000000000
H	3.169276985700	-3.169276985700	0.000000000000
Fe	0.000000000000	0.000000000000	0.000000000000
H	5.034528728000	1.350841842400	0.000000000000
H	-1.350841842400	5.034528728000	0.000000000000
H	-5.034528728000	-1.350841842400	0.000000000000
H	1.350841842400	5.034528728000	0.000000000000
H	-5.034528728000	1.350841842400	0.000000000000
H	-1.350841842400	-5.034528728000	0.000000000000
H	5.034528728000	-1.350841842400	0.000000000000
H	1.350841842400	-5.034528728000	0.000000000000

Cartesian coordinates of $^3A_{2g}$ electronic state of FeP optimized PBE0/def2-TZVP level of theory:

N	1.989807640600	0.000000000000	0.000000000000
N	0.000000000000	1.989807640600	0.000000000000
N	-1.989807640600	0.000000000000	0.000000000000
N	0.000000000000	-1.989807640600	0.000000000000
C	2.404256620600	2.404256620600	0.000000000000
C	-2.404256620600	2.404256620600	0.000000000000
C	-2.404256620600	-2.404256620600	0.000000000000
C	2.404256620600	-2.404256620600	0.000000000000
C	2.816536241800	1.087081515600	0.000000000000
C	-1.087081515600	2.816536241800	0.000000000000
C	-2.816536241800	-1.087081515600	0.000000000000
C	1.087081515600	2.816536241800	0.000000000000
C	-2.816536241800	1.087081515600	0.000000000000
C	-1.087081515600	-2.816536241800	0.000000000000
C	2.816536241800	-1.087081515600	0.000000000000
C	1.087081515600	-2.816536241800	0.000000000000
C	4.188854182100	0.676974418900	0.000000000000
C	-0.676974418900	4.188854182100	0.000000000000
C	-4.188854182100	-0.676974418900	0.000000000000
C	0.676974418900	4.188854182100	0.000000000000
C	-4.188854182100	0.676974418900	0.000000000000
C	-0.676974418900	-4.188854182100	0.000000000000
C	4.188854182100	-0.676974418900	0.000000000000
C	0.676974418900	-4.188854182100	0.000000000000
H	3.170181297800	3.170181297800	0.000000000000
H	-3.170181297800	3.170181297800	0.000000000000
H	-3.170181297800	-3.170181297800	0.000000000000

**H**	**3.170181297800**	**-3.170181297800**	**0.000000000000**
**Fe**	**0.000000000000**	**0.000000000000**	**0.000000000000**
**H**	**5.032946132100**	**1.350827150100**	**0.000000000000**
**H**	**-1.350827150100**	**5.032946132100**	**0.000000000000**
**H**	**-5.032946132100**	**-1.350827150100**	**0.000000000000**
**H**	**1.350827150100**	**5.032946132100**	**0.000000000000**
**H**	**-5.032946132100**	**1.350827150100**	**0.000000000000**
**H**	**-1.350827150100**	**-5.032946132100**	**0.000000000000**
**H**	**5.032946132100**	**-1.350827150100**	**0.000000000000**
**H**	**1.350827150100**	**-5.032946132100**	**0.000000000000**

Cartesian coordinates of $^5A_{1g}$ electronic state of FeP optimized PBE0/def2-TZVP level of theory:

**N**	**2.053997130250**	**0.000000000000**	**0.000000000000**
**N**	**0.000000000000**	**2.053997130250**	**0.000000000000**
**N**	**-2.053997130250**	**0.000000000000**	**0.000000000000**
**N**	**0.000000000000**	**-2.053997130250**	**0.000000000000**
**C**	**2.416615228928**	**2.416615228928**	**0.000000000000**
**C**	**-2.416615228928**	**2.416615228928**	**0.000000000000**
**C**	**-2.416615228928**	**-2.416615228928**	**0.000000000000**
**C**	**2.416615228928**	**-2.416615228928**	**0.000000000000**
**C**	**2.859028082406**	**1.097053808028**	**0.000000000000**
**C**	**-1.097053808028**	**2.859028082406**	**0.000000000000**
**C**	**-2.859028082406**	**-1.097053808028**	**0.000000000000**
**C**	**1.097053808028**	**2.859028082406**	**0.000000000000**
**C**	**-2.859028082406**	**1.097053808028**	**0.000000000000**
**C**	**-1.097053808028**	**-2.859028082406**	**0.000000000000**
**C**	**2.859028082406**	**-1.097053808028**	**0.000000000000**
**C**	**1.097053808028**	**-2.859028082406**	**0.000000000000**

C	4.234697250588	0.678721235699	0.000000000000
C	-0.678721235699	4.234697250588	0.000000000000
C	-4.234697250588	-0.678721235699	0.000000000000
C	0.678721235699	4.234697250588	0.000000000000
C	-4.234697250588	0.678721235699	0.000000000000
C	-0.678721235699	-4.234697250588	0.000000000000
C	4.234697250588	-0.678721235699	0.000000000000
C	0.678721235699	-4.234697250588	0.000000000000
H	3.183134570067	3.183134570067	0.000000000000
H	-3.183134570067	3.183134570067	0.000000000000
H	-3.183134570067	-3.183134570067	0.000000000000
H	3.183134570067	-3.183134570067	0.000000000000
Fe	0.000000000000	0.000000000000	0.000000000000
H	5.086641985290	1.343120820077	0.000000000000
H	-1.343120820077	5.086641985290	0.000000000000
H	-5.086641985290	-1.343120820077	0.000000000000
H	1.343120820077	5.086641985290	0.000000000000
H	-5.086641985290	1.343120820077	0.000000000000
H	-1.343120820077	-5.086641985290	0.000000000000
H	5.086641985290	-1.343120820077	0.000000000000
H	1.343120820077	-5.086641985290	0.000000000000

Cartesian coordinates of $^1B_{1g}$ electronic state of FeTBP optimized PBE0/def2-TZVP level of theory:

H	7.583214430456	-1.230402567755	0.000000000000
H	1.230402567755	7.583214430456	0.000000000000
H	-7.583214430456	1.230402567755	0.000000000000
H	-1.230402567755	7.583214430456	0.000000000000
H	-7.583214430456	-1.230402567755	0.000000000000

H	1.230402567755	-7.583214430456	0.000000000000
H	7.583214430456	1.230402567755	0.000000000000
H	-1.230402567755	-7.583214430456	0.000000000000
N	2.037745897275	0.000000000000	0.000000000000
N	0.000000000000	2.037745897275	0.000000000000
N	-2.037745897275	0.000000000000	0.000000000000
N	0.000000000000	-2.037745897275	0.000000000000
C	2.421702261999	-2.421702261999	0.000000000000
C	2.421702261999	2.421702261999	0.000000000000
C	-2.421702261999	2.421702261999	0.000000000000
C	-2.421702261999	-2.421702261999	0.000000000000
C	2.850641774499	-1.102092856524	0.000000000000
C	1.102092856524	2.850641774499	0.000000000000
C	-2.850641774499	1.102092856524	0.000000000000
C	-1.102092856524	2.850641774499	0.000000000000
C	-2.850641774499	-1.102092856524	0.000000000000
C	1.102092856524	-2.850641774499	0.000000000000
C	2.850641774499	1.102092856524	0.000000000000
C	-1.102092856524	-2.850641774499	0.000000000000
C	4.248610901064	-0.700369041067	0.000000000000
C	0.700369041067	4.248610901064	0.000000000000
C	-4.248610901064	0.700369041067	0.000000000000
C	-0.700369041067	4.248610901064	0.000000000000
C	-4.248610901064	-0.700369041067	0.000000000000
C	0.700369041067	-4.248610901064	0.000000000000
C	4.248610901064	0.700369041067	0.000000000000
C	-0.700369041067	-4.248610901064	0.000000000000

C	1.412081598246	-5.453096409046	0.000000000000
C	5.453096409046	1.412081598246	0.000000000000
C	-1.412081598246	5.453096409046	0.000000000000
C	-5.453096409046	1.412081598246	0.000000000000
C	-1.412081598246	-5.453096409046	0.000000000000
C	5.453096409046	-1.412081598246	0.000000000000
C	1.412081598246	5.453096409046	0.000000000000
C	-5.453096409046	-1.412081598246	0.000000000000
C	6.636938775304	-0.701056230907	0.000000000000
C	0.701056230907	6.636938775304	0.000000000000
C	-6.636938775304	0.701056230907	0.000000000000
C	-0.701056230907	6.636938775304	0.000000000000
C	-6.636938775304	-0.701056230907	0.000000000000
C	0.701056230907	-6.636938775304	0.000000000000
C	6.636938775304	0.701056230907	0.000000000000
C	-0.701056230907	-6.636938775304	0.000000000000
H	5.466499591810	-2.496453662866	0.000000000000
H	2.496453662866	5.466499591810	0.000000000000
H	-5.466499591810	2.496453662866	0.000000000000
H	-2.496453662866	5.466499591810	0.000000000000
H	-5.466499591810	-2.496453662866	0.000000000000
H	2.496453662866	-5.466499591810	0.000000000000
H	5.466499591810	2.496453662866	0.000000000000
H	-2.496453662866	-5.466499591810	0.000000000000
H	3.185843186359	-3.185843186359	0.000000000000
H	3.185843186359	3.185843186359	0.000000000000
H	-3.185843186359	3.185843186359	0.000000000000

H -3.185843186359 -3.185843186359 0.000000000000

Fe 0.000000000000 0.000000000000 0.000000000000

Cartesian coordinates of $^3A_{2g}$ electronic state of FeTBP optimized PBE0/def2-TZVP level of theory:

H 7.537066849999 -1.230788456558 0.000000000000

H 1.230788456558 7.537066849999 0.000000000000

H -7.537066849999 1.230788456558 0.000000000000

H -1.230788456558 7.537066849999 0.000000000000

H -7.537066849999 -1.230788456558 0.000000000000

H 1.230788456558 -7.537066849999 0.000000000000

H 7.537066849999 1.230788456558 0.000000000000

H -1.230788456558 -7.537066849999 0.000000000000

N 2.014138009110 0.000000000000 0.000000000000

N 0.000000000000 2.014138009110 0.000000000000

N -2.014138009110 0.000000000000 0.000000000000

N 0.000000000000 -2.014138009110 0.000000000000

C 2.407703170723 -2.407703170723 0.000000000000

C 2.407703170723 2.407703170723 0.000000000000

C -2.407703170723 2.407703170723 0.000000000000

C -2.407703170723 -2.407703170723 0.000000000000

C 2.827469089642 -1.098460447888 0.000000000000

C 1.098460447888 2.827469089642 0.000000000000

C -2.827469089642 1.098460447888 0.000000000000

C -1.098460447888 2.827469089642 0.000000000000

C -2.827469089642 -1.098460447888 0.000000000000

C 1.098460447888 -2.827469089642 0.000000000000

C 2.827469089642 1.098460447888 0.000000000000

C -1.098460447888 -2.827469089642 0.000000000000

C	4.212621318865	-0.698332199576	0.000000000000
C	0.698332199576	4.212621318865	0.000000000000
C	-4.212621318865	0.698332199576	0.000000000000
C	-0.698332199576	4.212621318865	0.000000000000
C	-4.212621318865	-0.698332199576	0.000000000000
C	0.698332199576	-4.212621318865	0.000000000000
C	4.212621318865	0.698332199576	0.000000000000
C	-0.698332199576	-4.212621318865	0.000000000000
C	1.412854965236	-5.408707134036	0.000000000000
C	5.408707134036	1.412854965236	0.000000000000
C	-1.412854965236	5.408707134036	0.000000000000
C	-5.408707134036	1.412854965236	0.000000000000
C	-1.412854965236	-5.408707134036	0.000000000000
C	5.408707134036	-1.412854965236	0.000000000000
C	1.412854965236	5.408707134036	0.000000000000
C	-5.408707134036	-1.412854965236	0.000000000000
C	6.591356542896	-0.701088891303	0.000000000000
C	0.701088891303	6.591356542896	0.000000000000
C	-6.591356542896	0.701088891303	0.000000000000
C	-0.701088891303	6.591356542896	0.000000000000
C	-6.591356542896	-0.701088891303	0.000000000000
C	0.701088891303	-6.591356542896	0.000000000000
C	6.591356542896	0.701088891303	0.000000000000
C	-0.701088891303	-6.591356542896	0.000000000000
H	5.421319436531	-2.496911739859	0.000000000000
H	2.496911739859	5.421319436531	0.000000000000
H	-5.421319436531	2.496911739859	0.000000000000

H	-2.496911739859	5.421319436531	0.000000000000
H	-5.421319436531	-2.496911739859	0.000000000000
H	2.496911739859	-5.421319436531	0.000000000000
H	5.421319436531	2.496911739859	0.000000000000
H	-2.496911739859	-5.421319436531	0.000000000000
H	3.173191677651	-3.173191677651	0.000000000000
H	3.173191677651	3.173191677651	0.000000000000
H	-3.173191677651	3.173191677651	0.000000000000
H	-3.173191677651	-3.173191677651	0.000000000000
Fe	0.000000000000	0.000000000000	0.000000000000

Cartesian coordinates of $^{5}A_{1g}$ electronic state of FeTBP optimized PBE0/def2-TZVP level of theory:

H	7.583474329142	-1.230327026961	0.000000000000
H	1.230327026961	7.583474329142	0.000000000000
H	-7.583474329142	1.230327026961	0.000000000000
H	-1.230327026961	7.583474329142	0.000000000000
H	-7.583474329142	-1.230327026961	0.000000000000
H	1.230327026961	-7.583474329142	0.000000000000
H	7.583474329142	1.230327026961	0.000000000000
H	-1.230327026961	-7.583474329142	0.000000000000
N	2.076759555258	0.000000000000	0.000000000000
N	0.000000000000	2.076759555258	0.000000000000
N	-2.076759555258	0.000000000000	0.000000000000
N	0.000000000000	-2.076759555258	0.000000000000
C	2.419407961118	-2.419407961118	0.000000000000
C	2.419407961118	2.419407961118	0.000000000000
C	-2.419407961118	2.419407961118	0.000000000000
C	-2.419407961118	-2.419407961118	0.000000000000

C	2.868321155479	-1.108183191879	0.000000000000
C	1.108183191879	2.868321155479	0.000000000000
C	-2.868321155479	1.108183191879	0.000000000000
C	-1.108183191879	2.868321155479	0.000000000000
C	-2.868321155479	-1.108183191879	0.000000000000
C	1.108183191879	-2.868321155479	0.000000000000
C	2.868321155479	1.108183191879	0.000000000000
C	-1.108183191879	-2.868321155479	0.000000000000
C	4.256812338570	-0.701202271286	0.000000000000
C	0.701202271286	4.256812338570	0.000000000000
C	-4.256812338570	0.701202271286	0.000000000000
C	-0.701202271286	4.256812338570	0.000000000000
C	-4.256812338570	-0.701202271286	0.000000000000
C	0.701202271286	-4.256812338570	0.000000000000
C	4.256812338570	0.701202271286	0.000000000000
C	-0.701202271286	-4.256812338570	0.000000000000
C	1.412162296771	-5.454055801842	0.000000000000
C	5.454055801842	1.412162296771	0.000000000000
C	-1.412162296771	5.454055801842	0.000000000000
C	-5.454055801842	1.412162296771	0.000000000000
C	-1.412162296771	-5.454055801842	0.000000000000
C	5.454055801842	-1.412162296771	0.000000000000
C	1.412162296771	5.454055801842	0.000000000000
C	-5.454055801842	-1.412162296771	0.000000000000
C	6.637823475208	-0.700477030903	0.000000000000
C	0.700477030903	6.637823475208	0.000000000000
C	-6.637823475208	0.700477030903	0.000000000000

C -0.700477030903 6.637823475208 0.000000000000

C -6.637823475208 -0.700477030903 0.000000000000

C 0.700477030903 -6.637823475208 0.000000000000

C 6.637823475208 0.700477030903 0.000000000000

C -0.700477030903 -6.637823475208 0.000000000000

H 5.466529304425 -2.496380959362 0.000000000000

H 2.496380959362 5.466529304425 0.000000000000

H -5.466529304425 2.496380959362 0.000000000000

H -2.496380959362 5.466529304425 0.000000000000

H -5.466529304425 -2.496380959362 0.000000000000

H 2.496380959362 -5.466529304425 0.000000000000

H 5.466529304425 2.496380959362 0.000000000000

H -2.496380959362 -5.466529304425 0.000000000000

H 3.185560752343 -3.185560752343 0.000000000000

H 3.185560752343 3.185560752343 0.000000000000

H -3.185560752343 3.185560752343 0.000000000000

H -3.185560752343 -3.185560752343 0.000000000000

Fe 0.000000000000 0.000000000000 0.000000000000

Table S1. The relative energies (kJ/mol) of excited states and contributions (in %) of electronic configurations to the wave functions from MCQDPT2 calculations.

State	Contributions	ΔE , kJ/mol	State	Contributions	ΔE, kJ/mol
	FeP			FeTBP	
${}^1B_{1g}$	$89[(e_g)^2(b_{2g})^2(a_{1g})^2(b_{1g})^0]$	107.5	${}^1B_{1g}$	$88[(e_g)^2(b_{2g})^2(a_{1g})^2(b_{1g})^0]$	
	$+7[(e_g)^2(b_{2g})^2(a_{1g})^1(b_{1g})^1]$			$+8[(e_g)^2(b_{2g})^2(a_{1g})^1(b_{1g})^1]$	119.0
${}^1B_{2g}$	$86[(e_g)^2(b_{2g})^2(a_{1g})^2(b_{1g})^0]$	107.5	${}^1B_{2g}$	$85[(e_g)^2(b_{2g})^2(a_{1g})^2(b_{1g})^0]$	
	$+6[(e_g)^2(b_{2g})^1(a_{1g})^2(b_{1g})^1]$			$+8[(e_g)^2(b_{2g})^1(a_{1g})^2(b_{1g})^1]$	120.6
${}^1E_g$	$91[(e_g)^3(b_{2g})^2(a_{1g})^1(b_{1g})^0]$	116.2	${}^1A_{1g}$	$86[(e_g)^4(b_{2g})^2(a_{1g})^0(b_{1g})^0]$	
				$+10[(e_g)^2(b_{2g})^2(a_{1g})^2(b_{1g})^0]$	120.6
${}^1A_{1g}$	$86[(e_g)^4(b_{2g})^2(a_{1g})^0(b_{1g})^0]$	117.1	${}^1E_g$	$91[(e_g)^3(b_{2g})^2(a_{1g})^1(b_{1g})^0]$	
	$+10[(e_g)^2(b_{2g})^2(a_{1g})^2(b_{1g})^0]$				123.1
${}^1A_{1g}$	$57[(e_g)^2(b_{2g})^2(a_{1g})^2(b_{1g})^0]$	186.1	${}^1A_{1g}$	$54[(e_g)^2(b_{2g})^2(a_{1g})^2(b_{1g})^0]$	
	$+25[(e_g)^2(b_{2g})^2(a_{1g})^1(b_{1g})^1]$			$+28[(e_g)^2(b_{2g})^2(a_{1g})^1(b_{1g})^1]$	
	$+10[(e_g)^4(b_{2g})^0(a_{1g})^2(b_{1g})^0]$			$+9[(e_g)^4(b_{2g})^0(a_{1g})^2(b_{1g})^0]$	
	$+6[(e_g)^4(b_{2g})^2(a_{1g})^0(b_{1g})^0]$			$+5[(e_g)^4(b_{2g})^2(a_{1g})^0(b_{1g})^0]$	194.6

$^1B_{2g}$	$71[(e_g)^4(b_{2g})^1(a_{1g})^1(b_{1g})^0]$	220.2	$^1B_{2g}$	$67[(e_g)^4(b_{2g})^1(a_{1g})^1(b_{1g})^0]$	
	$+26[(e_g)^2(b_{2g})^2(a_{1g})^1(b_{1g})^1]$			$+30[(e_g)^2(b_{2g})^2(a_{1g})^1(b_{1g})^1]$	224.1
^1E_g	$58[(e_g)^3(b_{2g})^1(a_{1g})^2(b_{1g})^0]$	232.6	^1E_g	$52[(e_g)^3(b_{2g})^1(a_{1g})^2(b_{1g})^0]$	
	$+29[(e_g)^1(b_{2g})^2(a_{1g})^2(b_{1g})^1]$			$+32[(e_g)^1(b_{2g})^2(a_{1g})^2(b_{1g})^1]$	
	$+6[(e_g)^3(b_{2g})^1(a_{1g})^1(b_{1g})^1]$			$+7[(e_g)^3(b_{2g})^1(a_{1g})^1(b_{1g})^1]$	239.2
$^1A_{1g}$	$77[(e_g)^2(b_{2g})^1(a_{1g})^2(b_{1g})^1]$	363.3	$^1B_{1g}$	$79[(e_g)^2(b_{2g})^1(a_{1g})^2(b_{1g})^1]$	
	$+9[(e_g)^4(b_{2g})^0(a_{1g})^2(b_{1g})^0]$			$+15[(e_g)^2(b_{2g})^2(a_{1g})^1(b_{1g})^1]$	
	$+5[(e_g)^0(b_{2g})^2(a_{1g})^2(b_{1g})^2]$				348.1
$^1B_{1g}$	$80[(e_g)^2(b_{2g})^1(a_{1g})^2(b_{1g})^1]$	367.5	$^1A_{1g}$	$77[(e_g)^2(b_{2g})^1(a_{1g})^2(b_{1g})^1]$	
	$+15[(e_g)^2(b_{2g})^2(a_{1g})^1(b_{1g})^1]$			$+8[(e_g)^4(b_{2g})^0(a_{1g})^2(b_{1g})^0]$	
				$+6[(e_g)^0(b_{2g})^2(a_{1g})^2(b_{1g})^2]$	351.1
$^1B_{2g}$	$79[(e_g)^2(b_{2g})^1(a_{1g})^2(b_{1g})^1]$	381.2	^1E_g	$89[(e_g)^3(b_{2g})^1(a_{1g})^1(b_{1g})^1]$	
	$+10[(e_g)^2(b_{2g})^2(a_{1g})^1(b_{1g})^1]$			$+7[(e_g)^3(b_{2g})^2(a_{1g})^0(b_{1g})^1]$	
	$+6[(e_g)^4(b_{2g})^1(a_{1g})^1(b_{1g})^0]$				357.7
^1E_g	$88[(e_g)^3(b_{2g})^1(a_{1g})^1(b_{1g})^1]$	382.5			
	$+10[(e_g)^3(b_{2g})^2(a_{1g})^0(b_{1g})^1]$				
$^3A_{2g}$	$96[(e_g)^2(b_{2g})^2(a_{1g})^2(b_{1g})^0]$	0	$^3A_{2g}$	$96[(e_g)^2(b_{2g})^2(a_{1g})^2(b_{1g})^0]$	0
^3E_g	$86[(e_g)^3(b_{2g})^2(a_{1g})^1(b_{1g})^0]$	15.8	^3E_g	$84[(e_g)^3(b_{2g})^2(a_{1g})^1(b_{1g})^0]$	
	$+12[(e_g)^3(b_{2g})^1(a_{1g})^2(b_{1g})^0]$			$+11[(e_g)^3(b_{2g})^1(a_{1g})^2(b_{1g})^0]$	11.1
$^3B_{2g}$	$86[(e_g)^4(b_{2g})^1(a_{1g})^1(b_{1g})^0]$	79.8	$^3B_{2g}$	$85[(e_g)^4(b_{2g})^1(a_{1g})^1(b_{1g})^0]$	
	$+12[(e_g)^2(b_{2g})^2(a_{1g})^1(b_{1g})^1]$			$+13[(e_g)^2(b_{2g})^2(a_{1g})^1(b_{1g})^1]$	71.5
^3E_g	$52[(e_g)^3(b_{2g})^1(a_{1g})^2(b_{1g})^0]$	137.7	^3E_g	$49[(e_g)^3(b_{2g})^1(a_{1g})^2(b_{1g})^0]$	
	$+27[(e_g)^3(b_{2g})^1(a_{1g})^1(b_{1g})^1]$			$+30[(e_g)^3(b_{2g})^1(a_{1g})^1(b_{1g})^1]$	
	$+11[(e_g)^1(b_{2g})^2(a_{1g})^2(b_{1g})^1]$			$+11[(e_g)^1(b_{2g})^2(a_{1g})^2(b_{1g})^1]$	
				$+5[(e_g)^3(b_{2g})^2(a_{1g})^1(b_{1g})^0]$	132.6
$^3B_{1g}$	$91[(e_g)^2(b_{2g})^1(a_{1g})^2(b_{1g})^1]$	264.1	$^3B_{1g}$	$90[(e_g)^2(b_{2g})^1(a_{1g})^2(b_{1g})^1]$	
	$+8[(e_g)^2(b_{2g})^2(a_{1g})^1(b_{1g})^1]$			$+9[(e_g)^2(b_{2g})^2(a_{1g})^1(b_{1g})^1]$	249.1
$^3B_{1g}$	$59[(e_g)^2(b_{2g})^1(a_{1g})^2(b_{1g})^1]$	275.7	^3E_g	$74[(e_g)^3(b_{2g})^1(a_{1g})^1(b_{1g})^1]$	
	$+27[(e_g)^2(b_{2g})^2(a_{1g})^1(b_{1g})^1]$			$+23[(e_g)^3(b_{2g})^2(a_{1g})^0(b_{1g})^1]$	
	$+13[(e_g)^4(b_{2g})^0(a_{1g})^1(b_{1g})^1]$				258.6
^3E_g	$86[(e_g)^3(b_{2g})^1(a_{1g})^1(b_{1g})^1]$	275.8	$^3B_{1g}$	$57[(e_g)^2(b_{2g})^1(a_{1g})^2(b_{1g})^1]$	
	$+8[(e_g)^1(b_{2g})^2(a_{1g})^2(b_{1g})^1]$			$+27[(e_g)^2(b_{2g})^2(a_{1g})^1(b_{1g})^1]$	
				$+14[(e_g)^4(b_{2g})^0(a_{1g})^1(b_{1g})^1]$	259.7
$^3B_{2g}$	$53[(e_g)^2(b_{2g})^2(a_{1g})^1(b_{1g})^1]$	282.1	$^3B_{2g}$	$53[(e_g)^2(b_{2g})^2(a_{1g})^1(b_{1g})^1]$	
	$+44[(e_g)^2(b_{2g})^1(a_{1g})^2(b_{1g})^1]$			$+45[(e_g)^2(b_{2g})^1(a_{1g})^2(b_{1g})^1]$	266.1
$^3A_{2g}$	$60[(e_g)^2(b_{2g})^2(a_{1g})^1(b_{1g})^1]$	284.0	$^3A_{2g}$	$64[(e_g)^2(b_{2g})^2(a_{1g})^1(b_{1g})^1]$	
	$+35[(e_g)^4(b_{2g})^1(a_{1g})^0(b_{1g})^1]$			$+29[(e_g)^4(b_{2g})^1(a_{1g})^0(b_{1g})^1]$	
				$+6[(e_g)^2(b_{2g})^1(a_{1g})^2(b_{1g})^1]$	267.0
$^5A_{1g}$	$100[(e_g)^2(b_{2g})^1(a_{1g})^2(b_{1g})^1]$	23.9	$^5A_{1g}$	$100[(e_g)^2(b_{2g})^1(a_{1g})^2(b_{1g})^1]$	8.5
^5E_g	$98[(e_g)^3(b_{2g})^1(a_{1g})^1(b_{1g})^1]$	46.0	^5E_g	$100[(e_g)^3(b_{2g})^1(a_{1g})^1(b_{1g})^1]$	26.7
$^5B_{2g}$	$100[(e_g)^2(b_{2g})^2(a_{1g})^1(b_{1g})^1]$	59.8	$^5B_{2g}$	$100[(e_g)^2(b_{2g})^2(a_{1g})^1(b_{1g})^1]$	42.8
$^5B_{1g}$	$100[(e_g)^2(b_{2g})^1(a_{1g})^1(b_{1g})^2]$	290.6	$^5B_{1g}$	$100[(e_g)^2(b_{2g})^1(a_{1g})^1(b_{1g})^2]$	261.8

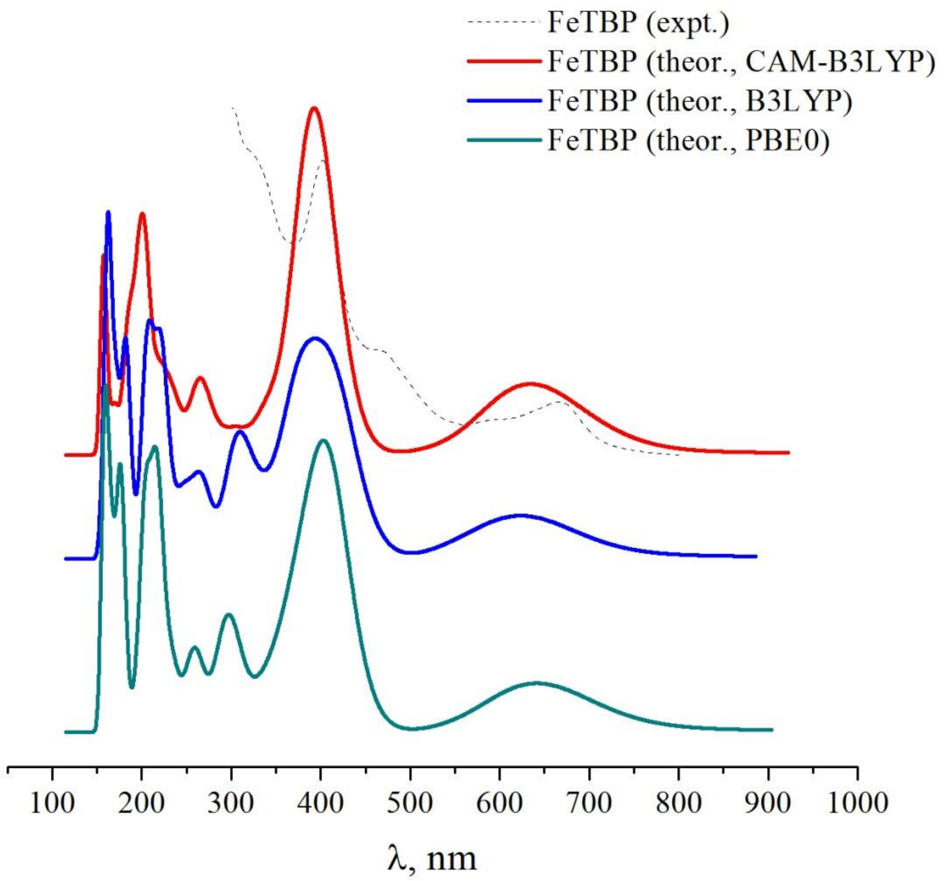

Figure S1. Normalized theoretical (sTDDFT) and experimental electronic absorption spectra of FeTBP in the DMF solvent. Gaussian broadening function with FWHM = 0.4 eV was used for the model spectra.
